# Combination of Remdesivir and Ivermectin Exerts Highly Potent and Synergistic Antiviral Activity Against Murine Coronavirus and SARS-CoV-2 Infections

**DOI:** 10.3390/cells15131146

**Published:** 2026-06-24

**Authors:** Ryan Z. Z. Lew, Douglas J. W. Tay, Jocelyn W. X. Ong, Jing Hui Low, Jing Liu, De Yun Wang, Justin J. H. Chu, Anand Kumar Andiappan, Kai Sen Tan, Vincent T. K. Chow

**Affiliations:** 1 Department of Microbiology and Immunology, Yong Loo Lin School of Medicine, National University of Singapore, Kent Ridge, Singapore 117545, Singapore; zzlew@u.nus.edu (R.Z.Z.L.); douglastay@nus.edu.sg (D.J.W.T.); miccjh@nus.edu.sg (J.J.H.C.); 2 NUS Medicine Biosafety Level 3 Core Facility, Yong Loo Lin School of Medicine, National University of Singapore, Kent Ridge, Singapore 117599, Singapore; 3 Infectious Diseases Translational Research Program, Yong Loo Lin School of Medicine, National University of Singapore, Kent Ridge, Singapore 117597, Singapore; entwdy@nus.edu.sg; 4 Singapore Immunology Network, Agency for Science, Technology and Research, Biopolis, Singapore 138648, Singapore; jocelyn_ong_wen_xin@immunol.a-star.edu.sg (J.W.X.O.); low_jing_hui@immunol.a-star.edu.sg (J.H.L.); anand_andiappan@immunol.a-star.edu.sg (A.K.A.); 5 Department of Otolaryngology, Yong Loo Lin School of Medicine, National University of Singapore, Kent Ridge, Singapore 119228, Singapore; entliuj@nus.edu.sg

**Keywords:** Remdesivir, Ivermectin, combination therapy, repurposed drugs, murine hepatitis virus, SARS-CoV-2, proteomics, transcriptomics, host pathways

## Abstract

The COVID-19 pandemic highlighted the urgent need to develop effective and broad-spectrum antiviral therapies against coronaviruses. One strategy to address this concern is a combination therapy using repurposed drugs against zoonotic viruses with pandemic potential. We previously demonstrated that the combination of Remdesivir and Ivermectin is highly potent and synergistic in inhibiting the replication of murine hepatitis virus (MHV) in RAW264.7 macrophages. This study investigated the interactions between the drug combination, coronavirus and host by proteomics and RNA sequencing of MHV-infected H2.35 murine liver epithelial cells. Time-of-addition and time-of-removal assays suggested that the drug combination likely affected the synthesis of viral RNA and viral protein. This combination drastically diminished the live virus titer greater than the respective monotherapies in MHV-infected H2.35 cells (by ~4 log_10_), as well as in SARS-CoV-2-infected VeroE6 cells and human nasal epithelial cells. Proteomic and transcriptomic analyses revealed that viral protein and RNA levels were significantly depressed upon combination treatment. The drug combination exhibited considerable negative effects upon host RNA processes and resulted in the upregulation of host protein processes (e.g., response to unfolded protein; protein insertion into ER membrane). Molecular pathways affected by the combination treatment were markedly distinct from the monotherapies and indicated that Ivermectin enhances Remdesivir by modulating critical host processes to synergistically exert its inhibitory effect on the coronavirus replication cycle.

## 1. Introduction

The severe acute respiratory syndrome coronavirus 2 (SARS-CoV-2) virus belongs to the *Betacoronavirus* subgenus in the coronavirus family and is the pathogen responsible for the COVID-19 pandemic. It is a single-stranded, positive-sense RNA virus that mainly causes respiratory illness in humans, although SARS-CoV-2 infection may also manifest enteric and neurologic symptoms [1].

The COVID-19 pandemic that emerged in 2019 highlighted major weaknesses in the global pandemic response pipeline. During the early pandemic phase, mitigation was limited mainly to legislative measures focusing on reducing social contact and limiting exposure to bodily fluids [2,3]. Consequently, the global economy was drastically impacted, culminating in huge economic and social losses [4,5].

While vaccines were undergoing development, alternative treatment strategies were necessary, and antivirals against SARS-CoV-2 were therefore a top research priority [6]. However, the development of de novo drugs requires about 8 to 10 years, with an average cost of 2.8 billion USD [7]. Moreover, only around 16% of drugs in development reach FDA approval [8]—hence, this conundrum urgently necessitated more immediate solutions. Among prospective solutions that received extensive attention during the pandemic were drug repurposing [6] and combination therapy [9].

Combination therapy refers to the use of at least two or more drugs in a cocktail as treatment. While introducing the possible risk of drug–drug interactions, it carries multiple advantages over monotreatment, e.g., the potential to reduce the drug concentration administered (thereby resulting in reduced overall costs and a lower burden on the patient) and the potential to hamper the development of antiviral resistance [9,10,11]. Selecting approved drugs for use in combination can also accelerate the approval process, since the safety profiles of these drugs are already known.

Drug repurposing refers to the process of screening approved drugs for new indications and inherently has several advantages over the development of novel drugs, i.e., reducing the financial cost of drug development and drastically shortening the time to clinic [12]. One major success of drug repurposing in the treatment of COVID-19 is Remdesivir, originally developed for its action against the Ebola virus disease [13]. Another example is Dexamethasone, a corticosteroid that has been shown to be synergistically beneficial when used clinically in combination [14].

Remdesivir is the prodrug form of a nucleoside analog that targets the RNA-dependent RNA polymerase (RdRp) of a wide range of RNA viruses and has been shown experimentally and clinically to be extremely potent against SARS-CoV-2 in vivo and in vitro [15,16,17,18,19]. Previous research in our laboratory revealed that Remdesivir acts synergistically with Ivermectin against murine hepatitis virus, or MHV [20]. MHV is a model betacoronavirus employed as a SARS-CoV-2 surrogate for in vitro infection of the RAW264.7 mouse macrophage cell line [20,21]. This significant synergy between these two drugs is corroborated by observations from other studies [22]. Although the antiviral effect of Remdesivir is attributed to its nucleoside analog activity, the mechanisms by which Ivermectin synergizes with Remdesivir to exert its inhibitory effects against coronaviruses remain unclear.

Ivermectin is currently used as a broad-spectrum antiparasitic agent that is effective against a wide range of parasites that plague livestock and humans, notably onchocerciasis [23,24]. Ivermectin is reported to exert broad-spectrum antiviral effects against flaviviruses via its activity on the NS3 viral helicase [25]. It also inhibits the dengue virus and human immunodeficiency virus via inhibition of host importin α/β dimerization [26,27]. Ivermectin has been shown to exhibit limited efficacy against SARS-CoV-2 in vitro [28], but prolonged ingestion at therapeutic doses (against onchocerciasis) may lead to toxicity [29]. Ivermectin’s mechanisms of action against SARS-CoV-2 in vitro have not been fully explored [30,31], except for in silico molecular docking experiments which suggest Ivermectin may interact with certain SARS-CoV-2 proteins [32]. Ivermectin was predicted to interact with the viral RdRp which coincides with the mechanism of Remdesivir. This research gap provides an excellent opportunity for a more targeted investigation. Collectively considered, Ivermectin represents an attractive target for drug repurposing and combination therapy since it is already approved for use in humans and has shown some efficacy against coronaviruses in vitro. In addition, the combination of Ivermectin with an appropriate antiviral such as Remdesivir may allow for lower doses to be administered and thus potentially decrease the incidence of adverse events.

Furthermore, other coronaviruses are of clinical concern, including severe acute respiratory syndrome coronavirus, or SARS-CoV [33], and Middle East respiratory syndrome coronavirus, or MERS-CoV [34], which are purported to be zoonotic [35] just like SARS-CoV-2 [1]. With animal reservoirs coming into ever-increasing contact with the human population due to the impact of urbanization, the risk of zoonotic illnesses will only increase and may culminate in the emergence of novel coronaviruses [36]. Therefore, effective broad-spectrum antivirals are needed as a first line of defense against coronaviruses.

The objectives of this study were to evaluate Remdesivir and Ivermectin as a combination treatment against MHV infection of H2.35 cells and SARS-CoV-2 infection of human nasal epithelial cells (hNECs). H2.35 is a murine epithelial-like cell line obtained from a primary hepatocyte isolate to mimic infection at epithelial surfaces [37]. H2.35 was selected as the target cell line for MHV to compare against our previous study of MHV infection of RAW264.7 macrophages [20] and also because SARS-CoV-2 can cause extrapulmonary infections, notably in the liver [38]. The hNECs are established from primary cells isolated from human nasal tissues and are differentiated via culture in the air–liquid interface (ALI), thus representing the primary infection site for SARS-CoV-2 [39]. This study aimed to identify the potential synergistic mechanisms of the Remdesivir–Ivermectin combination via virologic, host protein and mRNA analyses.

## 2. Materials and Methods

### 2.1. Cell Lines

The H2.35 cell line was derived from a tsA255-transformed hepatocyte culture isolated from a 6-week-old BALB/c female mouse. H2.35 cells were cultured in Dulbecco’s modified Eagle medium (DMEM) (HyClone, Logan, UT, USA) supplemented with 10% fetal bovine serum or FBS (Biowest, Nuaille, France) at 35 °C with 5% CO_2_ [20]. VeroE6 African green monkey kidney cells [33] expressing human transmembrane protease serine 2 (TMPRSS2) (BPS Bioscience, San Diego, CA, USA) were cultured in DMEM supplemented with 10% FBS and 3 µg/mL of puromycin at 37 °C with 5% CO_2_.

Human nasal epithelial stem/progenitor cells were derived from the nasal tissue of a 62-year-old female patient with septal deviation who underwent surgery at the National University Hospital, Singapore. The patient had no symptoms of respiratory infections and was not on corticosteroid treatment for at least 3 months. The specimen was deidentified immediately upon collection. Fully differentiated hNECs were obtained after air–liquid interface culture for 28 days. Ethical approval for the use of hNEC culture was obtained from the National University of Singapore Institutional Review Board (NUS-IRB-2020-33). Informed patient consent was obtained before sampling.

### 2.2. Virus Strains

Murine hepatitis virus strain A59 (MHV-A59, GenBank AY910861) was used as a representative betacoronavirus. Ancestral SARS-CoV-2 (hCoV-19/Singapore/26/2020, GSAID EPI_ISL_420103) and the SARS-CoV-2 Delta variant (hCoV-19/Singapore/1427/2021, GSAID EPI_ISL_2509080) were used in the SARS-CoV-2 experiments.

### 2.3. Drugs

Remdesivir (MedChemExpress, Monmouth Junction, NJ, USA) was prepared in aliquots of 10 mM in sterile water. Ivermectin (Merck, Burlington, MA, USA) was prepared to a concentration of 10 mM in dimethyl sulfoxide (DMSO) (Merck, Burlington, MA, USA). DMSO at 0.5% served as the drug vehicle control in all experiments.

### 2.4. MHV Infection of H2.35 Cells

The cell culture medium was removed from the confluent H2.35 cell monolayer before washing the cells twice with phosphate-buffered saline (PBS). Cells were infected with MHV at an MOI of 0.1, and attachment was allowed to proceed for 1 h at 35 °C with 5% CO_2_. The MHV inoculum was removed and replaced with DMEM supplemented with 2% FBS and 0.5% DMSO [20].

### 2.5. SARS-CoV-2 Infection of VeroE6 Cells and Human Nasal Epithelial Cells

The cell culture medium was removed from the confluent VeroE6 cell monolayer before washing the cells twice with PBS. VeroE6 cells were infected with SARS-CoV-2 at an MOI of 0.04 or 0.0004 for 1 h at 37 °C with 5% CO_2_, and the viral inoculum was removed.

Fully differentiated hNECs were washed once with Dulbecco’s phosphate-buffered saline (PBS) for 10 min at 37 °C with 5% CO_2_. The hNECs were infected apically with SARS-CoV-2 at an MOI of 0.1 for 1 h at 37 °C with 5% CO_2_. The viral inoculum was then removed.

### 2.6. Virus Plaque Assays

Ten-fold serial dilutions of supernatants from infection experiments were prepared for inoculation of H2.35 cells (for MHV) or VeroE6 cells (for SARS-CoV-2) in duplicates and incubated for 1 h at 35 °C (for MHV) and at 37 °C (for SARS-CoV-2) with 5% CO_2_. The MHV inoculum was replaced by a liquid overlay consisting of 1.2% Avicel (International Flavors and Fragrances, New York, NY, USA), 1 mM of HEPES (Gibco, Waltham, MA, USA), and 0.05% NaHCO_3_ (Gibco, Waltham, MA, USA) in MEM (Gibco, Waltham, MA, USA) and incubated for 72 h at 35 °C with 5% CO_2_. The SARS-CoV-2 inoculum was replaced by DMEM containing 1.2% microcrystalline cellulose (Sigma-Aldrich, Burlington, MA, USA), and incubated for 72 h at 37 °C with 5% CO_2_.

After 3 days of incubation, the liquid overlay was removed, and the cells were fixed with 4% (by volume in PBS) formaldehyde (Sigma-Aldrich, Burlington, MA, USA). The virus plaques were quantified by staining with 1% crystal violet (Sigma-Aldrich, Burlington, MA, USA), which was prepared by dissolving 1 g of crystal violet powder in 5 mL of absolute ethanol and diluting with 95 mL of 4% formaldehyde [20].

### 2.7. Half-Maximal Inhibitory Concentration (IC50)

H2.35 cells were seeded in 6-well plates at a density of 5 × 10^5^ cells per well and infected with MHV as described above. The respective drug was then added at the following concentrations: Ivermectin at 0 µM, 0.01 µM, 0.1 µM, 1 µM, 10 µM, and 100 µM and Remdesivir at 0 µM, 0.01 µM, 0.1 µM, 0.5 µM, 1 µM, and 10 µM. The samples were incubated for 24 h at 35 °C with 5% CO_2_, and the supernatants were collected to determine the virus titer by plaque assay. Prism 8.0.2 (GraphPad Software, San Diego, CA, USA) was utilized to plot a non-linear regression to determine the 50% inhibitory concentration (IC50) of each drug [20].

### 2.8. Cell Viability Assay, Half-Maximal Cytotoxic Concentration (CC50) and Selectivity Index

H2.35 and VeroE6 cells were seeded in 96-well plates at densities of 1.5 × 10^4^ and 3 × 10^4^ cells per well, respectively. Infection was performed, and combinations of Remdesivir and Ivermectin at different concentrations were added to the final cell culture medium. Infection was allowed to progress for 24 h at 35 °C with 5% CO_2_. Cell viability assay was then performed using the CellTiter 96 AQueous One Solution cell proliferation assay kit (Promega, Madison, WI, USA). Absorbance was measured at a wavelength of 490 nm using the Infinite 200 plate reader (Tecan, Mannedorf, Switzerland). Values were normalized to wells lacking both Remdesivir and Ivermectin. To calculate the 50% cytotoxic concentration (CC50), the values were used to plot a non-linear regression graph using Prism 8.0.2 (GraphPad Software, San Diego, CA, USA). The selectivity index of Remdesivir and Ivermectin was calculated according to the formula of CC50 divided by IC50 [20].

### 2.9. Checkerboard Assay and Bliss Synergy Score

H2.35 cells were seeded in 12-well plates at a density of 3 × 10^5^ cells per well and infected with MHV at an MOI of 0.1 for 1 h. Combinations of Remdesivir and Ivermectin at different concentrations were then added to the final cell culture medium. The drug concentrations tested were Remdesivir at 0 µM, 0.1 µM, 0.3 µM, and 0.5 µM and Ivermectin at 0 µM, 2 µM, 4 µM, and 6 µM. After 48 h of incubation, the supernatant from each well was then collected to determine the virus titer by plaque assay as described above. The Bliss synergy score was calculated from the values obtained from the checkerboard assay using SynergyFinder 3.0.

All SARS-CoV-2 experiments were conducted in the Biosafety Level 3 facility at the National University of Singapore. VeroE6 cells were seeded in 24-well plates (at 1.5 × 10^5^ cells per well) one day before SARS-CoV-2 infection was carried out at an MOI of 0.0004 for 1 h. Different concentrations of Remdesivir and/or Ivermectin (in combination or individually) were then added after the viral inoculum was removed. Cytopathic effect (CPE) was observed daily for up to 6 days (at which distinct CPE was evident in infected cells). CPE was scored for each well on a scale of 0 to 4 (0: no CPE, 1: ≤25% CPE, 2: >25% to ≤50% CPE, 3: >50% to ≤75% CPE, 4: >75% CPE).

To follow up on the checkerboard assay, VeroE6 cells were infected for 1 h at a higher MOI of 0.04 with SARS-CoV-2 (ancestral strain or Delta variant), and 2 µM of Remdesivir and/or 2 µM of Ivermectin (in combination or individually) was then introduced to the respective wells after the viral inoculum was removed. Supernatants were then harvested 48 h after the infection for virus plaque assay.

In addition, fully differentiated hNECs were also infected for 1 h at an MOI of 0.1 with the SARS-CoV-2 ancestral strain, and 2 µM of Remdesivir and/or 2 µM of Ivermectin (in combination or individually) was then introduced to the apical chamber after the viral inoculum was removed. Apical washes were then harvested 48 h after the infection for virus plaque assay.

### 2.10. Time-of-Addition (TOA) Assay

H2.35 cells were seeded in 96-well plates at a density of 1.5 × 10^4^ cells per well and infected with MHV as described above. At 0, 2, 4, 6, 8, 12, and 18 h post-infection, the initial cell culture medium in the respective well was replaced with DMEM supplemented with 2% FBS and 3 µM of Remdesivir. At 24 h post-infection, the supernatant was collected to determine the virus titer by plaque assay. This procedure was repeated for the 6 µM Ivermectin monotreatment, the combination treatment with 0.3 µM of Remdesivir and 6 µM of Ivermectin, or DMEM supplemented with 2% FBS with 0.5% DMSO (as a negative control).

### 2.11. Time-of-Removal (TOR) Assay

H2.35 cells were seeded in 96-well plates at a density of 1.5 × 10^4^ cells per well and infected with MHV as described above. The initial cell culture medium consisted of DMEM supplemented with 2% FBS and 3 µM of Remdesivir. At 0, 2, 4, 6, 8, 12, and 18 h post-infection, the cell culture medium in the respective well was then removed and replaced with DMEM supplemented with 2% FBS. At 24 h post-infection, the supernatant was collected to determine the virus titer by plaque assay. This procedure was repeated for the 6 µM Ivermectin monotreatment, the combination treatment with 0.3 µM of Remdesivir and 6 µM of Ivermectin, or DMEM supplemented with 2% FBS with 0.5% DMSO (as a negative control).

### 2.12. Reverse Transcription–Quantitative Polymerase Chain Reaction (RT-qPCR)

Viral RNA was harvested from each supernatant sample using the QIAamp MinElute virus spin kit (Qiagen, Hilden, Germany) following the manufacturer’s protocol. RT-qPCR was performed using the qScript cDNA synthesis kit (Quantabio, Beverly, MA, USA) according to the manufacturer’s protocol. The qPCR assay was performed using the LightCycler96 (Roche, Basel, Switzerland) under the following conditions: preincubation at 95 °C for 10 min; three-step amplification (50 cycles of 95 °C for 10 sec, 40 °C for 5 sec, 72 °C for 8 sec); and melt curve generation at 95 °C for 10 sec, 65 °C for 60 sec, and 97 °C for 1 sec. The forward and reverse primers used for amplifying the MHV N gene were 5′-ACGCTTACATTATCWACTTC-3′ and 5′-GATCTAAATTAGAATTGGTC-3′, respectively [20]. The GoTaq qPCR master mix (Promega, Madison, WI, USA) was utilized for the qPCR assay.

### 2.13. Proteomics by SWATH-MS

H2.35 cells were seeded in T25 flasks at a density of 6 × 10^5^ cells per flask (SPL Life Sciences, Pocheon, Republic of Korea) and infected with MHV as described above. Either 0.3 µM of Remdesivir, 6 µM of Ivermectin, a combination of 0.3 µM of Remdesivir and 6 µM of Ivermectin, 10 µM of Remdesivir (positive control), or DMEM supplemented with 2% FBS and 0.5% DMSO (negative control) was included in the final cell culture medium. After 24 h, cells were collected using a cell scraper (SPL Life Sciences, Pocheon, Republic of Korea) and pelleted. The cell pellet was then frozen at −80 °C until processed for sequential window acquisition of all theoretical mass spectra (SWATH-MS).

S-TRAP lysis buffer (50 mM of triethylammonium bicarbonate supplemented with 5% sodium dodecyl sulfate) was added to each cell pellet. Protein lysates were then collected and processed using the S-TRAP Micro column (ProtiFi, Fairport, NY, USA), according to the manufacturer’s protocol. Synthetic iRT peptides (Biognosys, Zurich, Switzerland) were added in each sample at a 10% final concentration for retention time alignment. Liquid chromatography (LC) was performed using the Waters ACQUITY UPLC M-Class system (Waters, Milford, MA, USA), utilizing Waters nanoEase M/Z Symmetry C18 (5 µm, 100 Å, 180 µm × 20 mm) as the trap column and Waters nanoEase M/Z Peptide BEH C18 (1.7 µm, 300 Å, 75 µm × 200 mm) as the analytical column. Solvent A (0.1% formic acid) and solvent B (0.1% formic acid in acetonitrile) were utilized with a flow rate of 300 nL/min. Mass spectrometry was performed on the elutes from LC using SCIEX TripleTOF 6600 (SCIEX, Marlborough, MA, USA) with the following ionization parameters: (a) nebulizer gas (GS1) was set at 10 units; (b) curtain gas at 30 units; (c) ionSpray voltage floating at 2200 V; and (d) interface heater temperature at 150 °C. SWATH-MS was then performed with the following parameters: (a) the precursor ion mass range was set at 400–1600 *m*/*z* with a 50 ms acquisition time; (b) a total of 100 variable SWATH windows were set across 400–1200 *m*/*z* (with a 1 Da window overlap and a minimum window width of 4 Da); (c) the mode was set to high sensitivity, with rolling energy collision enabled for each window with a 5 eV spread; and (d) the mass range was set to 100–1800 *m*/*z* with an accumulation time of 30 ms.

Data processing was performed using Spectronaut 18.0.230605.50606 (Biognosys, Zurich, Switzerland) via the DirectDIA workflow, utilizing the *Mus muculus* reference proteome (UP000000589, 2022_04 release, 55311 entries) from UniProt and the MHV-A59 reference proteome (UP000007192, 2022_04 release, 11 entries) from SwissProt. Common contaminant proteins were controlled via the inclusion of the common repository of adventitious proteins. Pulsar search was performed using the following settings: trypsin/P, fixed modification MMTS, variable modification acetyl (protein N-term) and oxidation (M), and a false discovery rate (FDR) filter of 0.01. DIA analysis was performed using a dynamic RT window and ion mass tolerance extraction with a summed peptide quantity from the top ten precursors, allowing for proteolytic peptides only. Retention time calibration was performed using Precision iRT (excluding deamidated peptides) and local (non-linear) regression. The global normalization strategy was performed on the median.

For pathway analysis, pairwise comparison (Kruskal–Wallis) with Spotfire 14.1 (TIBCO, Palo Alto, CA, USA) was used to determine differentially expressed proteins between the combination treatment and the other treatment conditions, i.e., 0.3 µM of Remdesivir (Rem03), 6 µM of Ivermectin (Iver6), infected (Inf), and uninfected (Uninf). Significant proteins from each comparison were then split into two groups depending on whether they were upregulated or downregulated in the combination treatment. Upregulated or downregulated proteins were then compared across pairwise comparisons such as (Combination versus 0.3 µM Remdesivir monotreatment) versus (Combination versus 6 µM Ivermectin monotreatment). These were then separated into three groups, e.g., based on whether they were upregulated in only one of the two pairwise comparisons or in both. Each group of proteins were then entered into Cytoscape 3.10.1 for StringDB pathway analysis. A graphical representation of the analysis process is depicted in Figure S2.

### 2.14. Transcriptomics by RNA Sequencing

H2.35 cells were infected with MHV and subjected to the various treatment conditions and controls. RNA was extracted using the mirVana miRNA isolation kit (Invitrogen, Carlsbad, CA, USA) according to the manufacturer’s protocol. Extracted RNA samples from the relevant experimental conditions and controls were subjected to bulk RNA sequencing.

The RNA samples were analyzed using the Agilent Bioanalyser (Agilent, Santa Clara, CA, USA) for quality assessment with an RNA integrity number (RIN) of 9.4 to 9.7. The cDNA libraries were prepared using 2 ng of total RNA via the Smart-seq2 protocol with the following modifications: the addition of 20 µM of TSO and using 200 pg of cDNA with 1/5 reaction of the Illumina Nextera XT kit (Illumina, San Diego, CA, USA). The length distribution of the cDNA libraries was monitored using a DNA High Sensitivity Reagent kit (Revvity, Waltham, MA, USA) on the Perkin Elmer Labchip. All samples were subjected to an indexed paired-end sequencing run of 2 × 151 cycles on the Illumina NovaSeq X sequencing system (Illumina, San Diego, CA, USA), targeting at least 15 million reads per sample.

FASTQ files were mapped to the mouse genome build GRCm38 using STAR. Gene counts were computed using featureCounts (part of the Subread package) using annotations from GENCODE (version M26). Differential gene expression analyses between combination treatment samples, other treatment samples and matched controls were performed using edgeR in a paired fashion under R (version 3.3.3). Multiple testing correction was performed using the method of Benjamini and Hochberg—genes with *p* values (false discovery rate, or FDR) less than 0.05 were considered to be significantly differentially expressed genes (DEGs).

For pathway analysis, DEGs from each comparison were obtained via edgeR as described above. The DEGs were then split into two groups depending on whether they were upregulated or downregulated in the combination treatment. Upregulated or downregulated DEGs were then compared across pairwise comparisons such as (Combination versus 0.3 µM Remdesivir) versus (Combination versus 6 µM Ivermectin). These were then separated into three groups, e.g., based on whether they were upregulated only in one of the two pairwise comparisons or in both. Each group of DEGs were then entered into Cytoscape 3.10.1 for StringDB pathway analysis. A graphical representation of the analysis process is depicted in Figure S2.

### 2.15. Quantification and Statistical Analyses

Statistical analyses were performed using PRISM 8.0.2 (GraphPad Software, San Diego, CA, USA) or Spotfire 14.1 (TIBCO, Palo Alto, CA, USA). The individual statistical tests and numbers of samples are included in the relevant figure legends and other sections where applicable.

## 3. Results

### 3.1. Combination Treatment with Remdesivir and Ivermectin Is Well-Tolerated by H2.35 Cells In Vitro

**Figure 1:**
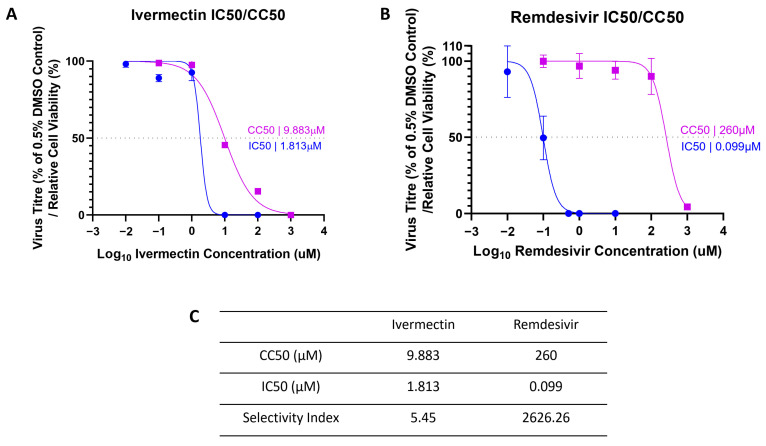
Pharmacological activity of Remdesivir and Ivermectin on H2.35 cells and MHV infection. (**A**) Graphs to determine half maximal inhibitory concentration (IC50) and 50% cytotoxic concentration (CC50) of Ivermectin (*n* = 3). (**B**) Graphs to determine IC50 and CC50 of Remdesivir (*n* = 3). (**C**) IC50 and CC50 values of Ivermectin and Remdesivir, with the calculated selectivity index (CC50 ÷ IC50).

The safety profiles of Remdesivir and Ivermectin as a monotherapy or in combination were first evaluated by cell viability assays. The CC50 values of Ivermectin and Remdesivir for H2.35 cells were 9.88 µM and 260 µM, respectively (Figure 1A and Figure 1B), indicating that Remdesivir is much better tolerated by H2.35 cells than Ivermectin. The IC50 values of Remdesivir and Ivermectin were then investigated to assess the effectiveness of the drugs as a monotherapy against MHV. The IC50 values of Ivermectin and Remdesivir were determined to be 1.813 µM and 0.099 µM, respectively (Figure 1). Remdesivir has a high selectivity index of 2626.26 (Figure 1C), suggesting that this drug is well-suited for clinical applications, as confirmed by its status as an FDA-approved drug. However, Ivermectin has a much lower selectivity index of 5.45 (Figure 1C), although this drug still retains a high safety margin for pharmacological intervention [40].

### 3.2. Combination of Remdesivir and Ivermectin Exhibits Remarkable Antiviral Synergism Against MHV Infection of H2.35 Cells

**Figure 2:**
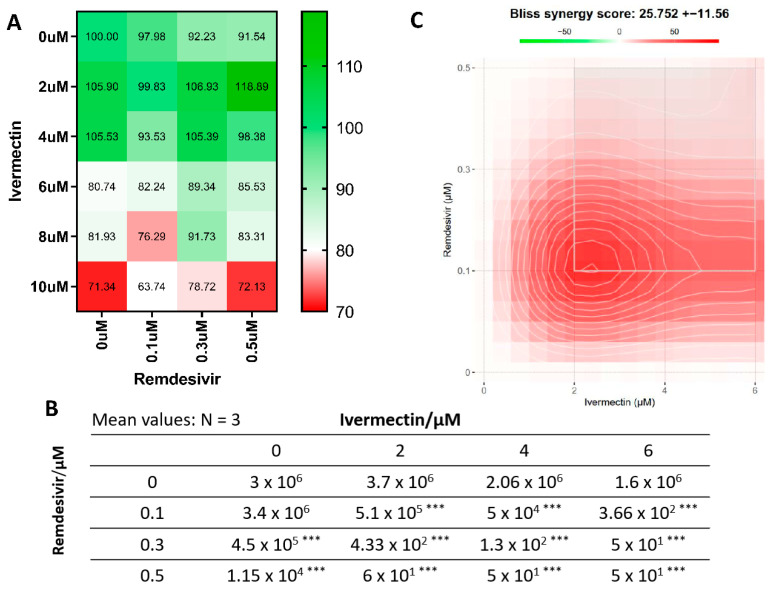
Checkerboard assay of inhibitory activity of Remdesivir and Ivermectin in combination or as monotreatment of H2.35 cells infected with MHV. (**A**) Cell viability assay of H2.35 cells subjected to monotreatments and different combinations of Remdesivir and Ivermectin (*n* = 3). (**B**) Checkerboard results of virus plaque assays. Remdesivir at 0.3 µM and Ivermectin at 6 µM were used as the constituent concentrations in the combination treatment in subsequent experiments (*** *p* < 0.001 compared to the untreated infected control; *n* = 3). (**C**) The Bliss synergy score plot was generated by SynergyFinder 3.0 from three independent experiments. As calculated by the Bliss algorithm, the dotted white square demarcates the most synergistic area with a score of 23.73, which indicates strong drug synergism.

Based on their respective IC50 values, a checkerboard assay was performed to evaluate the cell cytotoxicity effects of combinations of Remdesivir and Ivermectin at various concentrations. These drug combinations were generally well-tolerated by H2.35 cells. However, as concentrations of Ivermectin increased above 8 µM, cell viability decreased below the threshold of 80% (Figure 2A). In subsequent MHV infection experiments, the concentration of Ivermectin was therefore capped at 6 µM (with a cell viability of over 80%). In drug testing, a cell viability of 80% or greater is generally accepted for a drug to be considered as non-cytotoxic in accordance with international standards (ISO 10993-5:2009. Biological evaluation of medical devices. Part 5: Tests for in vitro cytotoxicity).

To determine the efficacy of the drug combination against MHV infection of H2.35 cells, checkerboard assay was performed with concentrations of Remdesivir ranging from 0 to 0.5 µM and Ivermectin from 0 to 6 µM. The combination therapy with 0.3 µM of Remdesivir plus 6 µM of Ivermectin attained a substantially greater reduction in live viral titer (4 to 5 log_10_) than the monotherapies, which could reduce viral titer by only ~1 log_10_ (0.3 µM of Remdesivir) (Figure 2B). Since the combination treatment could achieve a reduction in viral titer that was significantly greater than the sum of the individual drug treatments, this suggests that Remdesivir and Ivermectin act synergistically together to augment the antiviral effect [41]. Moreover, cell viability remained above 80% for all tested concentrations of Remdesivir in combination with Ivermectin at concentrations at or below 6 µM (Figure 2A).

Furthermore, the Bliss synergy score of 25.75 (Figure 2C) was considerably greater than 10, thus predicting that the combination is highly synergistic [42]. The summary synergy score refers to the average magnitude of the inhibitory effect that is in excess of the monotreatments [42]. The demarcated area in Figure 2C represents the most synergistic 3-by-3 doses in the dose–response matrix identified by the algorithm, coinciding with the statistically significant drug concentrations highlighted in Figure 2B. The most synergistic area score of 23.73 also indicated strong drug synergism.

In order to visualize the synergistic effect of the combination treatment, we examined monotherapies at concentrations which sub-optimally inhibit MHV in H2.35 cells. In addition, drug concentrations below their CC50 values and with acceptable cell viability when combined (≥80%) were selected for subsequent experiments, i.e., 0.3 µM of Remdesivir plus 6 µM of Ivermectin.

### 3.3. Combination of Remdesivir and Ivermectin Inhibits Post-Entry Stages of MHV Replication in H2.35 Cells

While Remdesivir’s mechanism of action (MOA) against coronavirus replication is well-documented [15,16,17,18], Ivermectin’s MOA against coronavirus infection is less clear, owing to its negligible antiviral effects as a monotherapy. Remdesivir is an adenosine analog that causes early termination of viral replication, whereas one proposed MOA of Ivermectin is by the inhibition of importin-α. Given that there is no known nuclear phase in the MHV replication cycle, it is thus important to consider other possible mechanisms to explain the synergism of this combination treatment.

**Figure 3:**
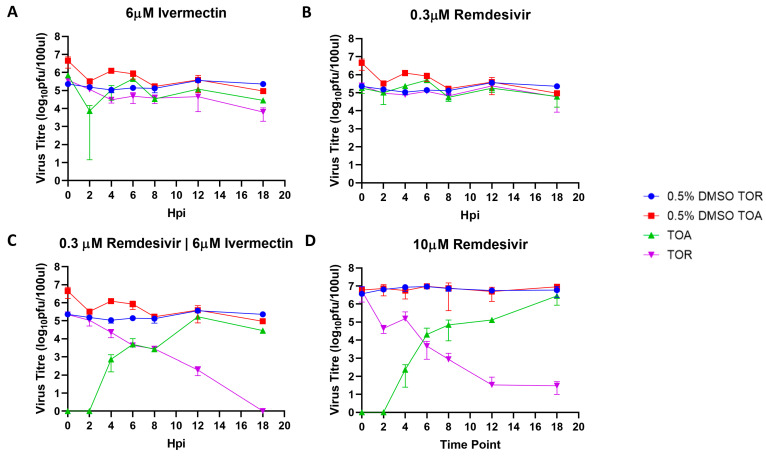
Time-of-addition and time-of-removal assays of monotreatment and combination treatment of Remdesivir and Ivermectin of MHV-infected H2.35 cells. (**A**) TOA/TOR results of monotherapy with 6 µM of Ivermectin (*n* = 3). (**B**) TOA/TOR results of monotherapy with 0.3 µM of Remdesivir (*n* = 3). (**C**) TOA/TOR results of combination treatment with 0.3 µM of Remdesivir plus 6 µM of Ivermectin (*n* = 3). (**D**) TOA/TOR results of monotherapy with 10 µM of Remdesivir, which served as a positive control for inhibition of viral replication (*n* = 3).

The TOA and TOR assays were thus performed for the selected concentrations of the individual drugs used in the combination treatment (Figure 3A,B), the combination treatment (Figure 3C), and 10 µM of Remdesivir as a positive control (Figure 3D). The monotreatment with the constituent drugs at the concentrations tested in the combination treatment were ineffective at reducing live viral titer—hence, the TOA and TOR curves did not give rise to a distinct intersection (Figure 3A,B). However, when Remdesivir was used at a sufficient concentration or in combination with Ivermectin, an intersection between the TOA and TOR curves was observed (Figure 3C,D).

An antiviral effect was exerted by high-dose Remdesivir within the 5 to 7 h time-frame (Figure 3D) and by the combination treatment within an extended time-frame of 5 to 9 h (Figure 3C), as determined by the intersection between their respective TOA and TOR plots. The time-frame of 10 µM of Remdesivir’s action coincides with the RNA synthesis phase of MHV replication [43,44]. With its extended intersection, the drug combination likely affects viral RNA synthesis as well as viral protein synthesis, which commences at about 5 to 6 h post-infection [44,45] or virion assembly. To further elucidate the host processes affected by the combination treatment, MHV-infected H2.35 cells were subjected to SWATH proteomics and bulk RNA sequencing.

### 3.4. Differences in Proteomes Indicate That the Combination Therapy Negatively Influences Viral Replication Through Modulation of Host RNA and Protein Processes

**Figure 4:**
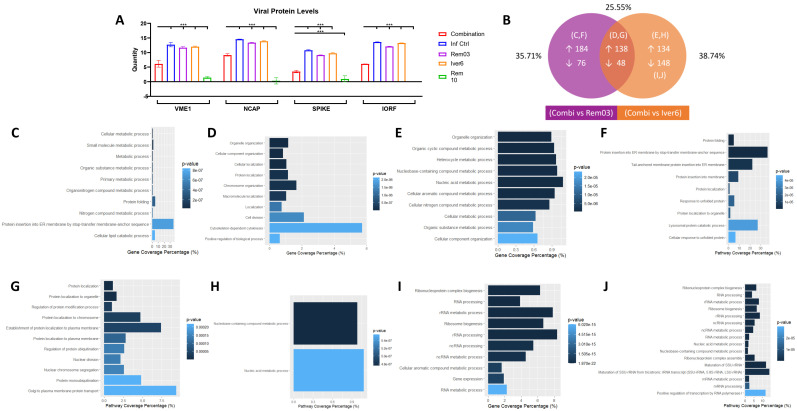
SWATH proteomics data processed via pathway analysis (*n* = 3). (**A**) Relative levels (in arbitrary units on the *y*-axis) of detected MHV proteins normalized to the uninfected control (set at 0) across different experimental conditions. VME1, membrane; NCAP, nucleocapsid; SPIKE, spike; IORF, N internal ORF protein (*** *p* < 0.001; *n* = 3). (**B**) Venn diagram showing percentages and numbers of differentially expressed proteins unique to either combination treatment versus 0.3 µM of Remdesivir (Combi vs. Rem03), combination treatment versus 6 µM of Ivermectin (Combi vs. Iver6), or similar in both comparisons. ↑ indicates upregulation, and ↓ indicates downregulation. The letters C to J correlate with the following figures to demarcate the set of proteins employed for pathway analysis. (**C**–**E**,**I**) Untargeted pathway analysis. (**F**–**H**,**J**) Targeted analysis of pathways selected by screening for regular expressions containing these terms: “RNA”, “ribo”, “nucle”, and “protein”. (**C**,**F**) Pathway analysis results of uniquely upregulated proteins in the Combi vs. Rem03 comparison when contrasted against the Combi vs. Iver6 comparison. (**D**,**G**) Pathway analysis results of upregulated proteins in both comparisons. (**E**,**H**) Pathway analysis results of uniquely upregulated proteins in the Combi vs. Iver6 comparison when contrasted against the Combi vs. Rem03 comparison. (**I**,**J**) Pathway analysis results of uniquely downregulated proteins in the Combi vs. Iver6 comparison when contrasted against the Combi vs. Rem03 comparison.

The combination treatment could reduce the levels of murine coronavirus proteins (membrane, nucleocapsid, spike, and the N internal ORF protein) compared to the infected control and its constituent monotherapies (Figure 4A). Moreover, the markedly diminished live virus titers and relatively lower viral RNA levels (Figure S1) by the combination of Remdesivir and Ivermectin corroborated that it was highly synergistic in inhibiting viral replication, in contrast to its monotherapies.

The proteomes of infected H2.35 cells with or without drug treatment were investigated to identify host pathways modulated by the combination treatment. Host proteins that were uniquely upregulated or downregulated in the combination condition of each pairwise comparison were subjected to pathway analysis according to the methodology illustrated in Figure S2. Figure 4B summarizes the percentages and numbers of upregulated or downregulated proteins unique to each comparison or common to both. It is noteworthy that most of the differentially expressed proteins (35.71% + 38.74%) were unique to either combination–monotreatment comparison, indicating that the drug combination influences a greater range of host processes compared to its monotherapies, which may contribute to its greater inhibitory effects against MHV. In support of this, the drug combination also yielded more differentially expressed proteins than the infected control compared to the uninfected control (Figure S3A).

The top ten most significant pathways from each pairwise comparison were examined and are depicted in Figure 4C–E,I. Upregulated pathways from all comparisons revealed an increased focus on metabolic processes in the drug combination compared to the monotherapies (Figure 4C–E). The drug combination downregulated pathways associated with host RNA processing when compared to the Ivermectin monotreatment (Figure 4I). This may be related to the Remdesivir component of the combination, which strongly inhibits viral RNA elongation.

Since the TOA and TOR assays suggested that viral RNA replication and viral protein synthesis were likely impacted by the drug combination, the associated host processes were targeted for further analysis by screening for the case insensitive regular expressions “RNA”, “ribo”, “nucle” and “protein” (Figure 4F–H,J). The upregulated host pathways of this targeted analysis revealed that the drug combination affected multiple host protein pathways (e.g., protein insertion into the endoplasmic reticulum membrane) to a greater extent compared to both monotherapies (Figure 4F,G). The set of proteins used to generate these pathways were not significantly different between the Ivermectin monotherapy and the combination therapy. This implies that Ivermectin may positively modulate host metabolic machinery by acting synergistically with Remdesivir to strongly perturb MHV replication, even though Ivermectin alone could not directly inhibit viral replication (Figure 4H). Downregulated pathways confirmed that host RNA processes were impacted by the drug combination compared to the Ivermectin monotherapy (Figure 4J). 

### 3.5. Differences in Transcriptomes Indicate That Combination Therapy Negatively Influences Viral Replication Through Increased Modulation of Host RNA Processes

**Figure 5:**
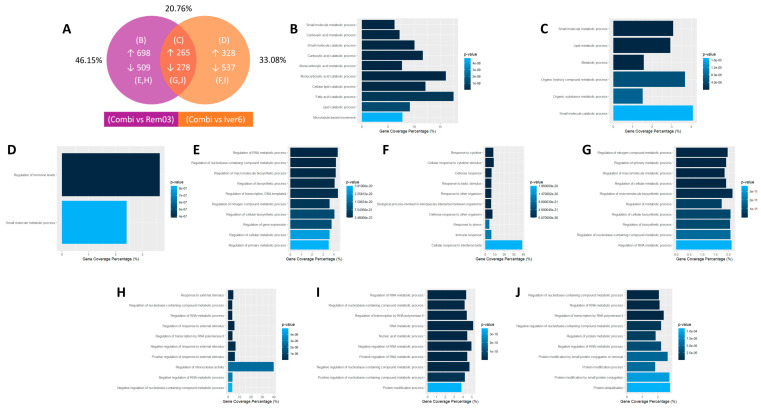
Bulk RNA sequencing data processed via pathway analysis (*n* = 3). (**A**) Venn diagram showing percentages and numbers of differentially expressed genes unique to either Combi vs. Rem03 or Combi vs. Iver6 or similar in both comparisons. ↑, upregulation; ↓, downregulation. The letters B to J correlate with the following figures to demarcate the set of genes employed for pathway analysis. (**B**–**G**) Untargeted pathway analysis. (**H**–**J**) Targeted analysis of pathways selected by screening for regular expressions containing these terms: “RNA”, “ribo”, “nucle”, and “protein”. (**B**) Pathway analysis results of uniquely upregulated genes in the Combi vs. Rem03 comparison when contrasted against the Combi vs. Iver6 comparison. (**C**) Pathway analysis results of upregulated genes in both comparisons. (**D**) Pathway analysis results of uniquely upregulated genes in the Combi vs. Iver6 comparison when contrasted against the Combi vs. Rem03 comparison. (**E**,**H**) Pathway analysis results of uniquely downregulated genes in the Combi vs. Rem03 comparison when contrasted against the Combi vs. Iver6 comparison. (**F**,**I**) Pathway analysis results of uniquely downregulated genes in the Combi vs. Iver6 comparison when contrasted against the Combi vs. Rem03 comparison. (**G**,**J**) Pathway analysis results of downregulated genes in both comparisons.

The transcriptomes of infected H2.35 cells with or without drug treatment were investigated to unravel host pathways impacted by the combination treatment. Host mRNAs that were uniquely upregulated or downregulated by the combination condition of each pairwise comparison were subjected to pathway analysis according to the methodology depicted in Figure S2. Figure 5A summarizes the percentages and numbers of upregulated or downregulated gene transcripts unique to each pairwise comparison or common to both. A larger proportion of unique differentially expressed genes (46.15%) was found in the drug combination than in the Remdesivir monotreatment. In line with the proteomics data, most of the differentially expressed genes (46.15% + 33.08%) were unique to either combination–monotreatment comparison. This higher contribution of differentially expressed genes in the combination treatment was also observed in relation to the infected control compared to the uninfected control (Figure S4A).

The top ten most significant pathways from each comparison were analyzed and are visualized in Figure 5B–G. Congruent with the proteomics data, upregulated pathways revealed a focus on metabolic and catabolic processes in the drug combination compared to the monotherapies (Figure 5B–D). Pathways downregulated by the combination treatment compared to the Remdesivir monotherapy were focused on the regulation of biosynthetic processes (Figure 5E). Pathways downregulated by the combination treatment compared to the Ivermectin monotherapy were focused on the immune and interferon responses (Figure 5F). Pathways downregulated by the combination treatment compared to both monotherapies were mainly focused on the regulation of metabolic and biosynthetic processes (Figure 5G). The regulation of two of these pathways (i.e., nucleobase-containing compound metabolic process and RNA metabolic process) was also identified in the corresponding proteomics data. These findings may be reflected by the significantly diminished viral titer in the combination group compared to the monotherapies.

Associated host processes were targeted by screening for the case-insensitive regular expressions (“RNA”, “ribo”, “nucle” and “protein”) similar to the proteomics analysis (Figure 5H–J). The focused analysis revealed an absence of upregulated pathways containing these keywords. Conversely, the filtered downregulated pathways were focused on RNA, nucleic acid and protein metabolic processes as well as the regulation of ribonuclease activity (Figure 5H–J). Notably, the theme of these downregulated pathways was congruent with the pathways downregulated in the drug combination compared to the infected control (Figure S4F,G).

### 3.6. Combination of Remdesivir and Ivermectin Is Strongly Synergistic in Inhibiting SARS-CoV-2 Infection of VeroE6 Cells and Human Nasal Epithelial Cells

**Figure 6:**
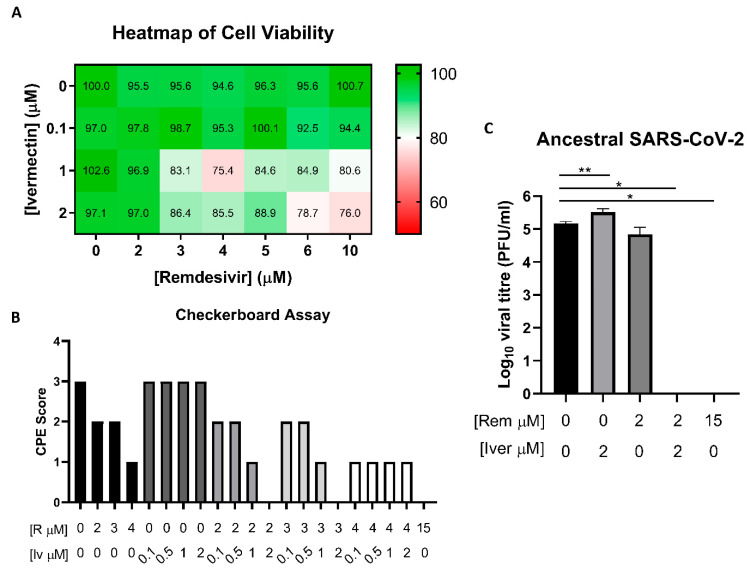
Checkerboard assay for effects of Remdesivir and Ivermectin treatments at different concentrations on SARS-CoV-2 infection. (**A**) VeroE6 cell viability assays of combinations of Remdesivir and Ivermectin at different concentrations (*n* = 3). (**B**) Checkerboard assay to determine effects of different combinations of Remdesivir (R) and Ivermectin (Iv) on cytopathic effect (CPE) scores of VeroE6 cells infected with ancestral SARS-CoV-2 strain (*n* = 3). CPE was scored in each well on a scale of 0 to 4 (0, no CPE; 1, ≤25% CPE; 2, >25% to ≤50% CPE; 3, >50% to ≤75% CPE; 4, >75% CPE). Representative images of CPE are shown in Figure S5A. Using SynergyFinder 3.0, the most synergistic area score was calculated by the Bliss algorithm to be 11.11, indicating drug synergism. (**C**) Virus plaque assay results of treatment with Remdesivir (Rem) and/or Ivermectin (Iver) in combination or as monotreatment on SARS-CoV-2-infected VeroE6 cells. The combination of 2 µM of Remdesivir and 2 µM of Ivermectin achieved complete viral inhibition, similar to the 15 µM of Remdesivir treatment which served as the positive control. One-way ANOVA with Dunnet’s correction was used to evaluate the statistical significance (* *p* < 0.05; ** *p* < 0.01; *n* = 3).

To ensure that the antiviral efficacy of the drug combination against betacoronaviruses was not restricted to MHV, we proceeded to evaluate it against the medically important SARS-CoV-2. We assessed the cytotoxicity of Remdesivir and Ivermectin on VeroE6 cells as single drugs or in combination (Figure 6A). VeroE6 cells were viable and could tolerate Remdesivir and Ivermectin as single drugs up to their concentrations of 5 µM and 2 µM, respectively. However, when given in combination, cell viability diminished with higher concentrations of Remdesivir (≥4 µM) with Ivermectin (≥1 µM).

Thus, we selected a range of 0 to 4 µM of Remdesivir with 0 to 2 µM of Ivermectin for the checkerboard assay to identify the optimal concentrations of the drug combination for inhibiting SARS-CoV-2 (Figure 6B and Figure S5A). VeroE6 cells were infected with ancestral SARS-CoV-2 at an MOI of 0.0004, followed by treatment with either Remdesivir or Ivermectin alone, or in combination, and observed for cytopathic effect (CPE) for 6 days. Remdesivir, a known inhibitor of SARS-CoV-2, resulted in a dose-dependent decrease in CPE when administered alone. In contrast, administration of Ivermectin alone was not inhibitory to SARS-CoV-2. However, we observed that the combination of 2 µM of Remdesivir with 2 µM of Ivermectin and 3 µM of Remdesivir with 2 µM of Ivermectin resulted in complete inhibition of SARS-CoV-2 (no CPE), similar to 15 µM of Remdesivir as a positive control (Figure 6B). Therefore, we selected the combination of 2 µM of Remdesivir with 2 µM of Ivermectin for further evaluation, which includes a lower dose of Remdesivir and high cell viability (97%), indicating minimal toxicity.

We next compared the effects of the monotherapies versus combination-drug treatment in reducing live SARS-CoV-2 titers (ancestral strain and Delta variant) in VeroE6 cells at a higher MOI of 0.04. While the monotreatments involving 2 µM of Remdesivir or 2 µM of Ivermectin did not significantly inhibit SARS-CoV-2, the drug combination achieved complete viral inhibition of the ancestral strain even at the higher MOI tested (5 log_10_ reduction in live virus, *p* = 0.02; Figure 6C). This combination also inhibited the SARS-CoV-2 Delta variant by a 3.7-log_10_ reduction in live virus (Figure S5B). Live virus inhibition (2.5 log_10_ reduction) was also achieved by this combination treatment of primary differentiated human nasal epithelial cells infected with ancestral SARS-CoV-2 at a higher MOI of 0.1 (Figure S5D). Taken together, our results indicate that Remdesivir augmented with Ivermectin can confer synergistic inhibition of the ancestral and Delta variants of SARS-CoV-2, including authentic infection of human nasal epithelial cells.

The concentration of 2 µM of Remdesivir used in our experiments is within the half-maximal effective concentration (EC50) of Remdesivir against VeroE6 cells infected with ancestral SARS-CoV-2, i.e., ranging from 0.77 to 23.15 µM in previous studies [22,46].

## 4. Discussion

Although Remdesivir, as an approved treatment against SARS-CoV-2, has great efficacy against coronaviruses when used as a monotreatment, we have shown that the addition of Ivermectin to Remdesivir confers enhanced antiviral activity against both SARS-CoV-2 and MHV. Irrespective of the animal lineage of the cells (i.e., human nasal cells, monkey VeroE6 cells, or mouse H2.35 or RAW264.7 cells) and the coronavirus strain (i.e., SARS-CoV-2 or MHV) investigated in our in vitro experiments, the combination of Remdesivir and Ivermectin consistently maintains its strong synergistic effect against coronaviruses, indicative of broad-spectrum activity. This combination also facilitates the usage of much reduced concentrations of Remdesivir, thus minimizing the chances of toxicity. The TOA and TOR assays further suggest that the combination treatment impacts viral transcription and translation during the coronavirus replication cycle. In addition, proteomic and transcriptomic analyses indicate that the drug combination affects host protein processes compared to the Remdesivir monotreatment, thereby contributing to its remarkable synergy against coronaviruses. These findings suggest that the combination of Remdesivir and Ivermectin is a promising broad-spectrum antiviral cocktail that is efficacious against different members of the *Betacoronavirus* subgenus.

While vaccines may be able to prevent the spread of SARS-CoV-2, viral escape mutants that evade the antibodies generated upon vaccination have been observed [1]. Furthermore, Remdesivir-resistant variants of SARS-CoV-2 have developed naturally from in vivo studies and in vitro experiments and highlight the distinct possibility of Remdesivir resistance with successive passages [47,48]. A combination therapy may be able to address this issue by impeding the development of resistance [9,10], further supporting the use of a combination therapy against coronaviruses as a complementary approach to vaccines for combatting acute infections.

Although the synergism of Remdesivir and Ivermectin against coronaviruses has also been previously reported [22], we have further proceeded to deconvolute the viral and host processes involved in the observed synergistic effects of the combination treatment. We have shown that the drug combination exerts its effects on post-entry viral processes, with the likely processes impacted being viral transcription, viral RNA replication and translation based on the time-frame at which the combination treatment exhibits its inhibitory effects [43,44,45]. This finding is further corroborated by the proteomic and transcriptomic data, where both viral protein and RNA levels were depressed upon combination treatment.

In addition to viral protein and RNA levels, several key host pathways associated with protein and RNA metabolism were impacted by the combination treatment. Given that the virus exploits host machinery to engage in viral protein synthesis and RNA replication, the combination treatment could induce alterations in these host pathways and their related processes which ultimately perturb the viral life-cycle. Crucially, host pathways related to the generation of new RNA molecules were negatively affected in both the proteome and transcriptome analyses, which could hinder viral RNA replication. Host pathways related to protein folding and localization were positively affected by the drug combination.

Unsurprisingly, there was some, but incomplete, overlap between the pathways revealed by the transcriptomic and proteomic analyses, which is a well-documented phenomenon, especially in humans who have the lowest mRNA/protein concordance values [49]. Notwithstanding this, the overall theme of the top ten most significant downregulated host pathways was that the combination treatment culminated in downregulation of host RNA processes compared to the monotreatment conditions.

While Remdesivir represses viral transcription and viral RNA replication, our proteomic findings indicate that the drug combination is likely to additionally impact viral translation, which leads to synergistic perturbation of the viral life-cycle. Relevant host protein pathways affected by the drug combination over the Remdesivir monotreatment include “protein folding”, “protein insertion into membrane” and “response to unfolded protein”. Our proteomics data on the combination versus Remdesivir monotherapy (0.3 µM) comparison reveal that the host unfolded protein response (UPR) pathway was upregulated by the drug combination. This is interesting given that the combination treatment resulted in considerably reduced live virus titers and lower viral RNA levels. This finding warrants future detailed investigations to elucidate the molecular effects of the combination treatment on host UPR and other pathways modulated by viral infection [50]. Interestingly, host protein pathways associated with a normal functioning cell were upregulated by the drug combination, reflecting the antiviral efficacy of the combination treatment in restoring normal cellular physiologic mechanisms since they appear to have compensated for MHV’s modulation of the host protein processes.

Previous studies on coronavirus infections have alluded to the involvement of the P21-activated kinase 1 (PAK1) protein as one of the known molecular mechanisms of action of Ivermectin [51]. PAK1 is a serine/threonine kinase involved in a variety of essential cellular functions, including but not limited to cell motility, metabolism, stress, proliferation, and survival [52]. SARS-CoV-2 infection is associated with the upregulation of actin cytoskeleton remodeling via the RhoA pathway [53]. PAK1 mediates cytoskeletal rearrangement and promotes SARS-CoV-2 entry. PAK inhibition can suppress SARS-CoV-2 infection, reduce lung viral load, and alleviate pulmonary inflammation in infected Syrian hamsters [54]. Notably, Ivermectin is associated with diminished PAK1 expression via the ubiquitination-mediated degradation pathway [55]. Congruent with this, the proteomics data reveal that the PAK1 protein was reduced by the Ivermectin monotherapy and by the combination treatment compared to the infected control (Figure S6).

MHV uses the N-terminal domain of its spike protein to bind to its receptor, the carcinoembryonic antigen-related cell adhesion molecule 1 (CEACAM1), which is an intercellular adhesion glycoprotein associated with the actin cytoskeleton [56]. Associations between plasma membrane proteins and the actin cytoskeleton play critical roles in facilitating key cell–cell contacts which are subject to exquisite regulation. The localization of CEACAM1 at cell–cell boundaries is regulated by the Rho family of GTPases, i.e., Rac1 and Cdc42, which direct CEACAM1 to cell-to-cell contacts. PAK acts downstream of these two GTPases, and activated PAK also targets CEACAM1 to cell–cell contacts [57,58,59]. Through cell fusion, MHV can also induce and exploit the formation of syncytia to aid its spread to neighboring susceptible cells [60]. Hence, PAK inhibition is expected to perturb cell–cell contacts mediated by CEACAM1, which is associated with the actin cytoskeleton.

In this context, there are some similarities between MHV and SARS-CoV-2. The SARS-CoV-2 receptor ACE2 is localized at the apical cell–cell junctions of epithelial cells [61]. During SARS-CoV-2 infection, host heparan sulfate can stimulate spike-induced ACE2 receptor clustering, cell–cell fusion as well as syncytium formation [62].

Another role of PAK1 is its interaction and colocalization with the signal transducer and activator of transcription 3 (STAT3) in the nucleus. The PAK1/STAT3 complex binds to the interleukin-6 (*IL-6*) promoter and regulates *IL-6* gene transcription. PAK1 inhibition decreases nuclear pSTAT3 and extracellular IL-6 levels [63,64]. This is congruent with our previous finding that the combination treatment of MHV-infected macrophages with Remdesivir and Ivermectin significantly decreased *IL-6* expression [20].

Clinical trials have generally reported that Ivermectin did not affect the outcomes of COVID-19 patients [65], although one randomized controlled trial showed that Ivermectin treatment could result in a statistically significant lower viral load in patients with mild-to-moderate COVID-19 [66]. Notwithstanding this, the combination of Remdesivir and Ivermectin may be promising as a broad-spectrum antiviral therapy against betacoronaviruses and appears to retain its synergistic efficacy across different cell types (whether human, monkey or mouse, or of respiratory epithelial, hepatocyte, macrophage or kidney origin). Importantly, the drug combination permits the use of reduced concentrations of both drugs while maintaining robust antiviral activity against betacoronaviruses.

One limitation of this study is the lineage of the H2.35 cell line, which is derived from murine hepatocytes. Hence, the proteomic and transcriptomic responses detailed in this study may be limited in part to the liver. Nonetheless, we have shown that the synergism of Remdesivir and Ivermectin is not solely restricted to a specific host cell type, i.e., its synergistic effect is consistent across different cell types and even animal species.

Another limitation is the relatively lower MOI of 0.1 of MHV used for in vitro infection of cells and that the drug combination was added 1 h after infection. The rationale for using a lower MOI was to mimic the gradual progression of viral infection observed in most patients. Hence, it would be interesting to conduct future experiments at higher MOIs as well as at various time-points before and after infection to compare treated cells versus untreated cells.

In this study, cell viability assays were performed at 24 h of drug treatment. Future experiments are warranted to evaluate cell viability after longer periods of the combination treatment and to measure other parameters of cellular function, such as cell cycle distribution.

Vero monkey kidney cells generally express high levels of the P-glycoprotein (Pgp) efflux transporter pumps of drug compounds. Ivermectin is known to inhibit Pgp, which may potentially increase the intracellular concentration of Remdesivir and thus cause a “synergistic” effect of the combination treatment. To address this potential mechanism of Ivermectin, future studies could evaluate the drug combination using the VeroE6 Pgp knockout cell line for a comparison with the wild-type VeroE6 cells [67,68]. However, the Human Protein Atlas (https://www.proteinatlas.org/ENSG00000085563-ABCB1/tissue/nasopharynx—accessed on 22 March 2026) and our H2.35 cell proteome data (under tested conditions) reveal that P-glycoprotein (ABCB1) was not detected in human nasopharyngeal epithelial cells nor in H2.35 murine hepatocytes, respectively. The absence of Pgp in hNECs and in H2.35 hepatocytes indicates that the Pgp-associated mechanism of Ivermectin is not relevant in these two cell types.

## 5. Conclusions

The current study has generated encouraging preliminary data on the efficacy of the relevant drug combination against SARS-CoV-2 infection of hNECs. Going forward, further rigorous and comprehensive analyses in vitro and in vivo should be performed for comparison with the findings from the MHV infection of H2.35 cells. These include drug safety, toxicity and efficacy studies in animal models as well as proteomics and transcriptomics of the combination treatment in the presence and absence of infection. The latter would determine whether the drug combination affects protein and gene expression in uninfected cells. The analyses may also be further expanded to other drug combinations and coronaviruses of interest, such as MERS-CoV. Additional investigations into the molecular interactions of Ivermectin that contribute to the observed synergism are warranted to fully elucidate the underlying molecular mechanisms. Future in vitro and in vivo investigations into any emergence of viral escape mutants when MHV or SARS-CoV-2 infection is subjected to the combination treatment are also crucial to ascertain the molecular mechanisms and clinical feasibility of this approach for treating coronavirus infections.

## Data Availability

Proteomics data from this study have been deposited in the PRoteomics IDEntifications Database (PRIDE) with the following identifier: PXD073706. Bulk RNA sequencing data have been deposited in the Gene Expression Omnibus (GEO) database with the following identifier: GSE317360.

## Supplementary Materials

The following supporting information can be downloaded at: https://www.mdpi.com/article/10.3390/cells15131146/s1, Figure S1: Results of virus plaque assays and corresponding RT-qPCR analyses showing viral titers of samples subjected to proteomics and RNA sequencing; Figure S2: Data analysis pipeline of proteomics and transcriptomics data leading to pathway analysis via Cytoscape; Figure S3: SWATH proteomics data processed via pathway analysis; Figure S4: Bulk RNA sequencing data processed via pathway analysis; Figure S5: Effects of Remdesivir and/or Ivermectin treatments on SARS-CoV-2 infection of VeroE6 cells and human nasal epithelial cell cultures; Figure S6: Relative quantity of the p21-activated kinase 1 (PAK1) protein in MHV-infected H2.35 cells subjected to different drug treatments as detected by proteomics analysis.
