# Supplementary material for: Combination of Remdesivir and Ivermectin Exerts Highly Potent and Synergistic Antiviral Activity Against Murine Coronavirus and SARS-CoV-2 Infections
Source: Cells. 2026 Jun 24;15(13):1146. doi: 10.3390/cells15131146 (PMC13359856; doi:10.3390/cells15131146)
Supplement: Supplementary file 1 [file cells-15-01146-s001.zip › cells-4327781-supplementary.pdf]

Supplementary Material

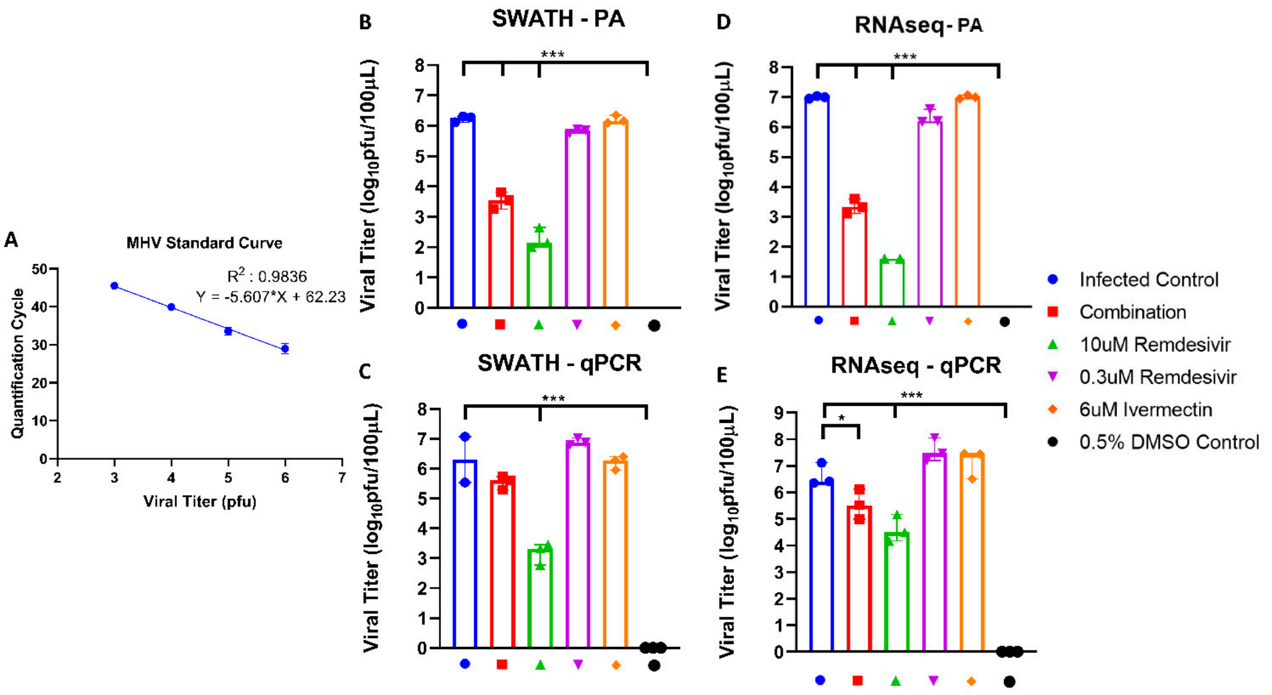

Supplementary Figure S1. Results of virus plaque assays and corresponding RT-qPCR analyses showing viral titers of samples subjected to proteomics and RNA sequencing.

(**A**) Standard curve constructed by plotting quantification cycle of the MHV nucleocapsid gene by RT-qPCR (*y*-axis) against known live viral titers of MHV in $\log_{10}$ PFU per 100 µL (*x*-axis) ($n = 3$). (**B**) Virus plaque assay (PA) results of samples subjected to SWATH proteomics (*** $p < 0.001$, $n = 3$). (**C**) Viral RNA loads detected by RT-qPCR assays of RNAs extracted from supernatants of samples subjected to SWATH proteomics (*** $p < 0.001$, $n = 3$). The equivalent live viral titers (*y*-axis) were extrapolated from the standard curve shown in panel A. (**D**) Virus plaque assay (PA) results of supernatants of samples subjected to bulk RNA sequencing (*** $p < 0.001$, $n = 3$). (**E**) Viral RNA loads detected by RT-qPCR assays of RNAs extracted from supernatants of samples subjected to bulk RNA sequencing (* $p = 0.0492$, *** $p < 0.001$, $n = 3$). The equivalent live viral titers (*y*-axis) were extrapolated from the standard curve shown in panel A. Statistical significance of the results were determined by one-way ANOVA with Dunnet's correction.

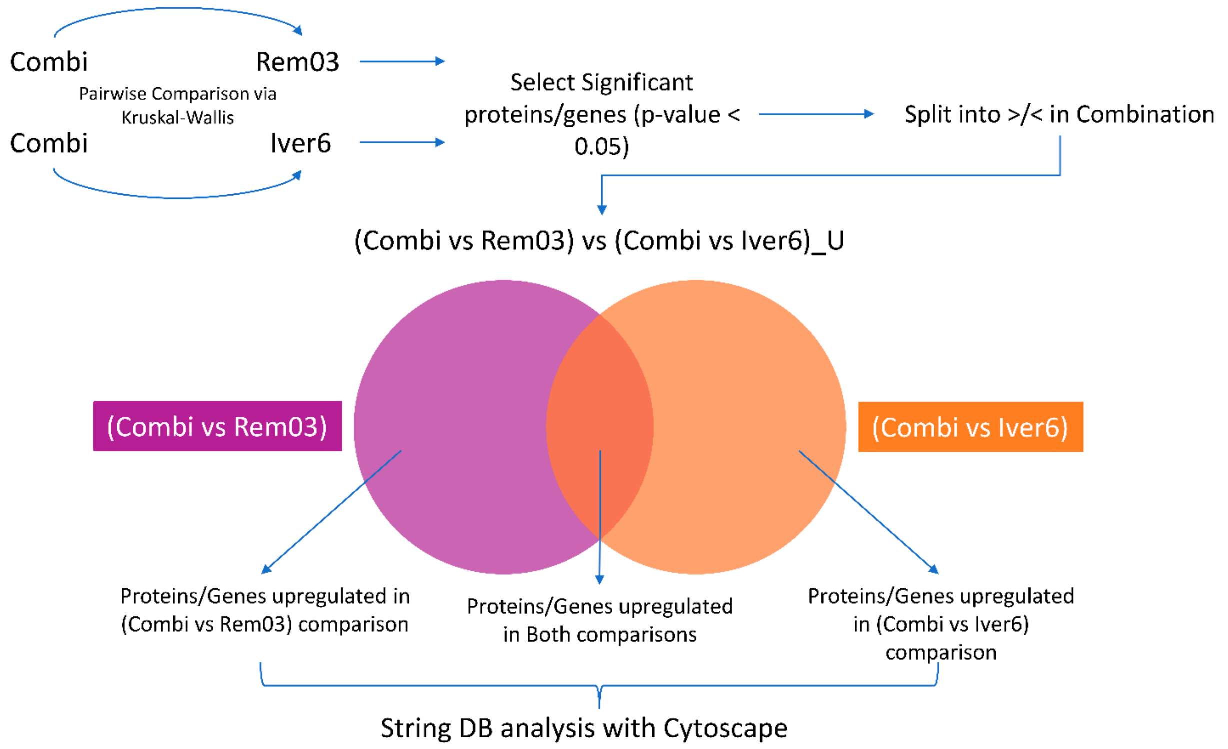

Supplementary Figure S2. Data analysis pipeline of proteomics and transcriptomics data leading to pathway analysis via Cytoscape.

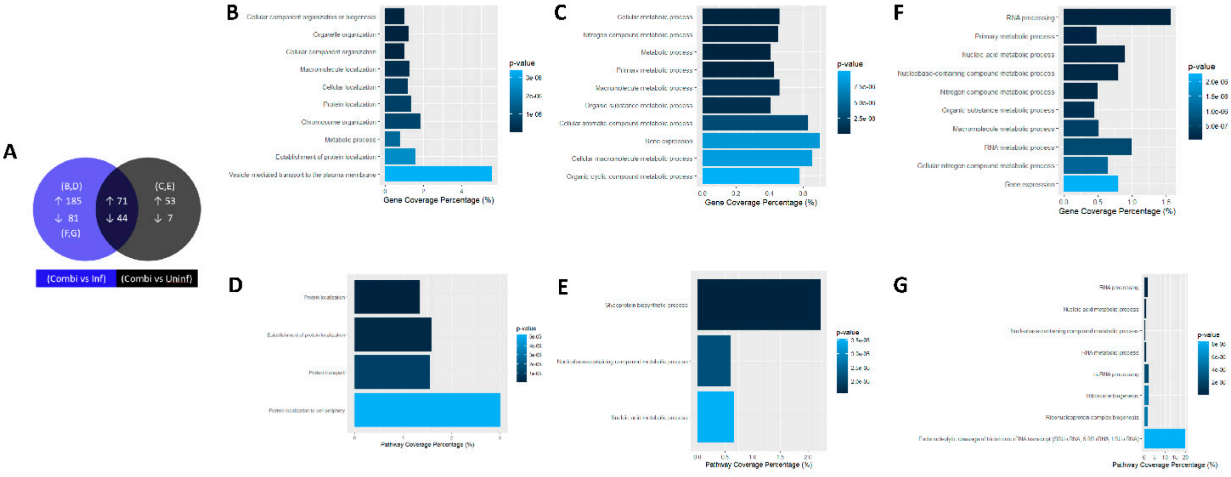
A
(B,D)
↑ 185
↓ 81
(F,G)
↑ 71
↓ 44
(C,E)
↑ 53
↓ 7
(Combi vs Inf)
(Combi vs Uninf)
B
C
F
D
E
G

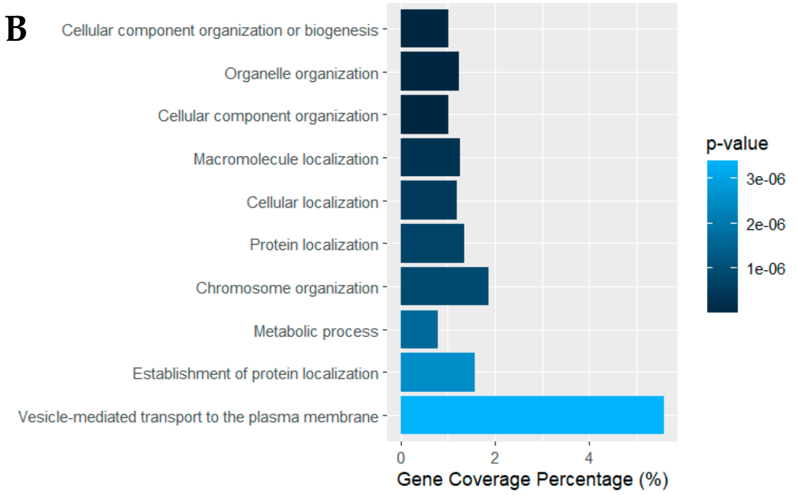
B
Cellular component organization or biogenesis
Organelle organization
Cellular component organization
Macromolecule localization
Cellular localization
Protein localization
Chromosome organization
Metabolic process
Establishment of protein localization
Vesicle-mediated transport to the plasma membrane
0
2
4
Gene Coverage Percentage (%)
p-value
3e-06
2e-06
1e-06

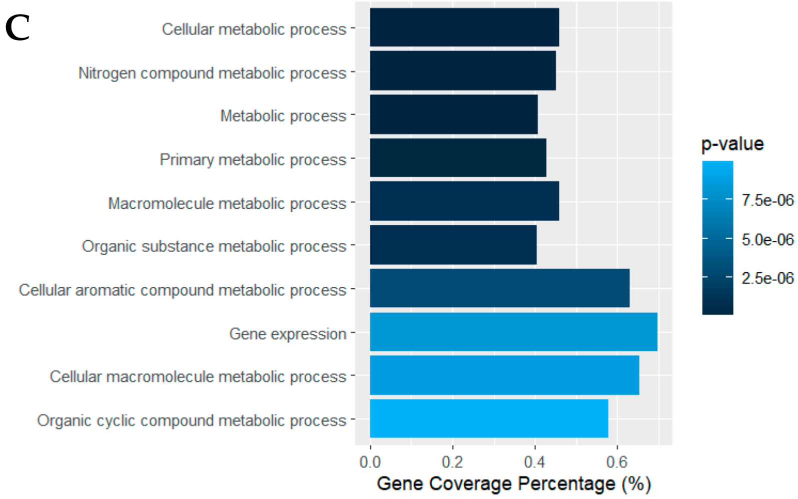
C
Cellular metabolic process
Nitrogen compound metabolic process
Metabolic process
Primary metabolic process
Macromolecule metabolic process
Organic substance metabolic process
Cellular aromatic compound metabolic process
Gene expression
Cellular macromolecule metabolic process
Organic cyclic compound metabolic process
0.0
0.2
0.4
0.6
Gene Coverage Percentage (%)
p-value
7.5e-06
5.0e-06
2.5e-06

D

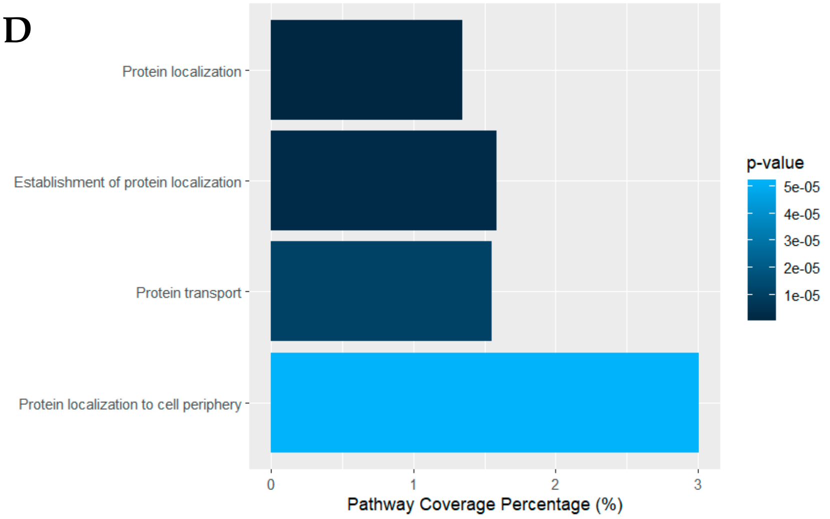
Protein localization
Establishment of protein localization
Protein transport
Protein localization to cell periphery
0
1
2
3
Pathway Coverage Percentage (%)
p-value
5e-05
4e-05
3e-05
2e-05
1e-05

E

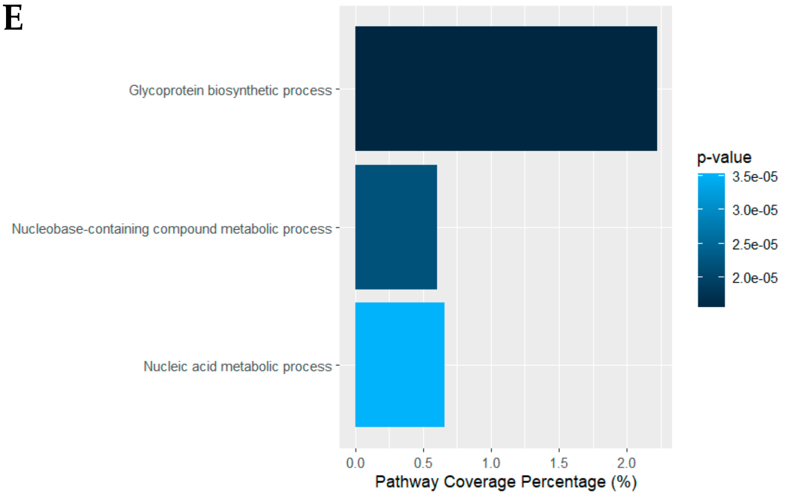
Glycoprotein biosynthetic process
Nucleobase-containing compound metabolic process
Nucleic acid metabolic process
0.0
0.5
1.0
1.5
2.0
Pathway Coverage Percentage (%)
p-value
3.5e-05
3.0e-05
2.5e-05
2.0e-05

F

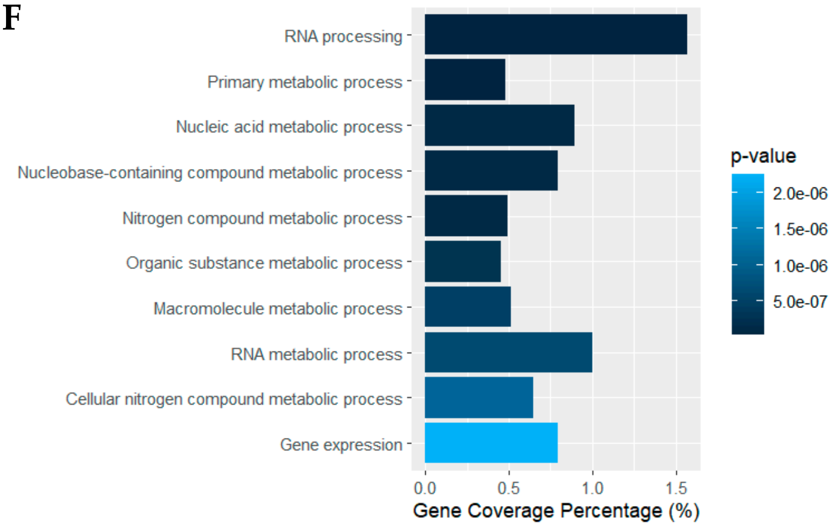
RNA processing
Primary metabolic process
Nucleic acid metabolic process
Nucleobase-containing compound metabolic process
Nitrogen compound metabolic process
Organic substance metabolic process
Macromolecule metabolic process
RNA metabolic process
Cellular nitrogen compound metabolic process
Gene expression
0.0
0.5
1.0
1.5
Gene Coverage Percentage (%)
p-value
2.0e-06
1.5e-06
1.0e-06
5.0e-07

G

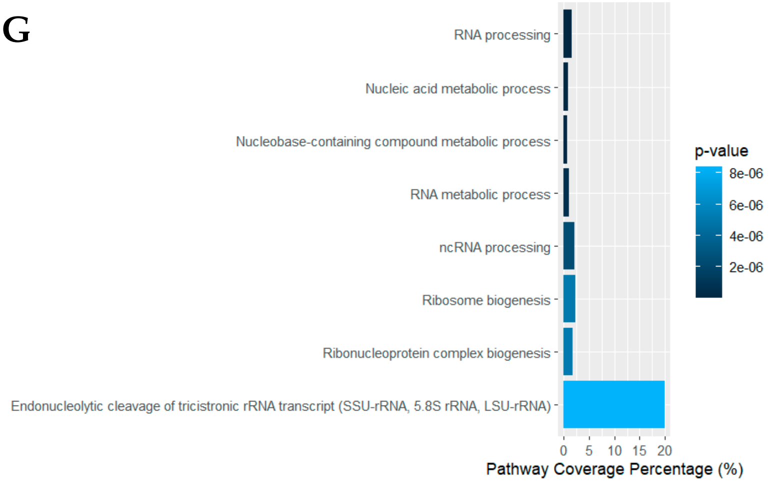

Supplementary Figure S3. SWATH proteomics data processed via pathway analysis.

(**A**) Venn diagram showing numbers of differentially expressed proteins unique to either combination treatment versus infected control (Combi versus Inf), combination treatment versus uninfected control (Combi versus Uninf), or similar in both comparisons. ↑, upregulation; ↓, downregulation. The letters **B** to **G** correlate with the following figures to demarcate the set of proteins employed for pathway analysis. (**B, C, F**) Untargeted pathway analysis. (**D, E, G**) Targeted analysis of pathways selected by screening for regular expressions containing these terms: "RNA", "ribo", "nucle", "protein". (**B, D**) Pathway analysis results of uniquely upregulated proteins in the Combi versus Inf comparison when contrasted against the Combi versus Uninf comparison. (**C, E**) Pathway analysis results of uniquely upregulated proteins in the Combi versus Uninf comparison when contrasted against the Combi versus Inf comparison. (**F, G**) Pathway analysis results of uniquely downregulated proteins in the Combi versus Inf comparison when contrasted against the Combi versus Uninf comparison.

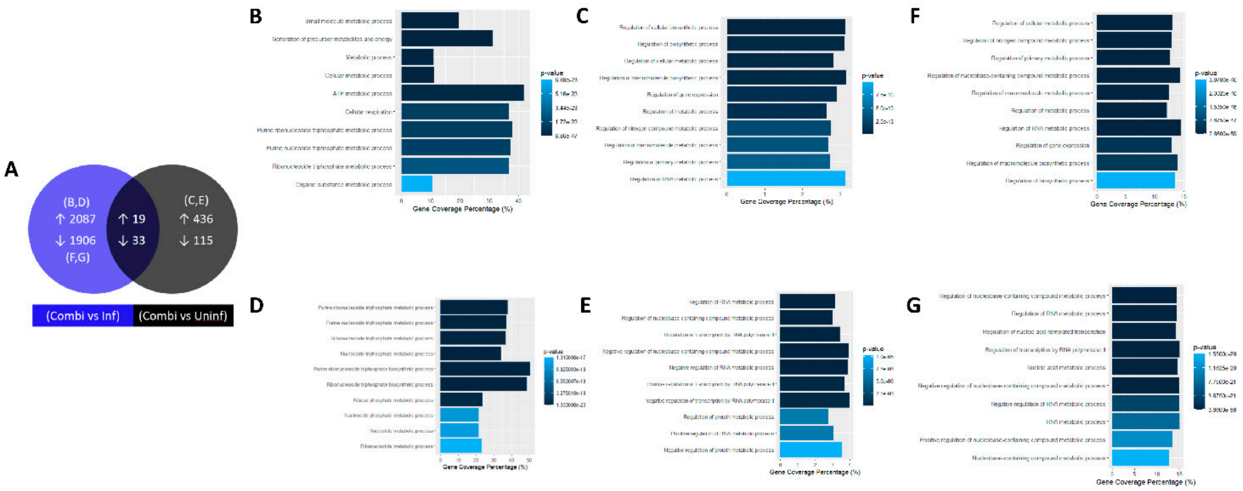
A
B
C
F
D
E
G
(B,D)
↑ 2087
↓ 1906
(F,G)
↑ 19
↓ 33
(C,E)
↑ 436
↓ 115
(Combi vs Inf)
(Combi vs Uninf)
Gene Coverage Percentage (%)
p-value

B

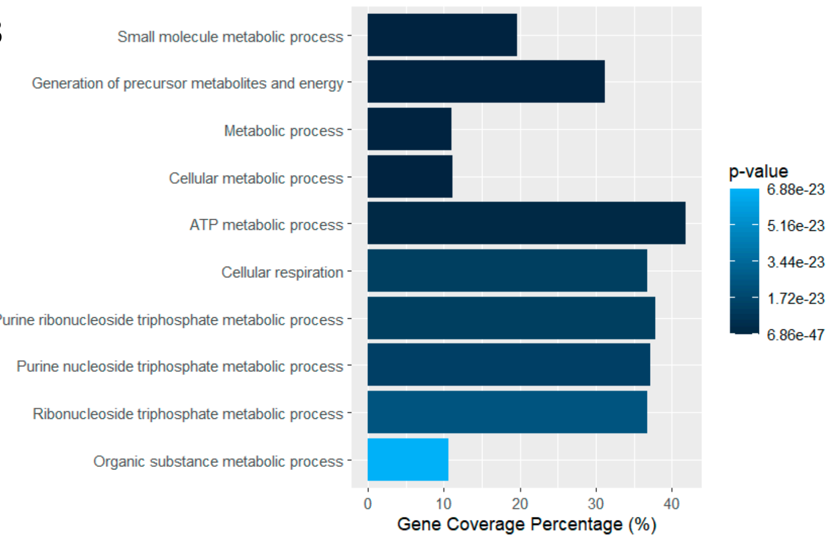
Small molecule metabolic process
Generation of precursor metabolites and energy
Metabolic process
Cellular metabolic process
ATP metabolic process
Cellular respiration
Purine ribonucleoside triphosphate metabolic process
Purine nucleoside triphosphate metabolic process
Ribonucleoside triphosphate metabolic process
Organic substance metabolic process
0
10
20
30
40
Gene Coverage Percentage (%)
p-value
6.88e-23
5.16e-23
3.44e-23
1.72e-23
6.86e-47

C

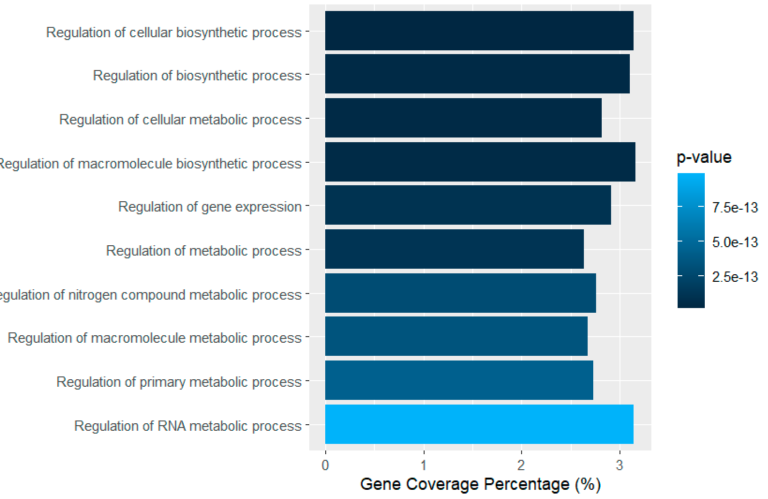
Regulation of cellular biosynthetic process
Regulation of biosynthetic process
Regulation of cellular metabolic process
Regulation of macromolecule biosynthetic process
Regulation of gene expression
Regulation of metabolic process
Regulation of nitrogen compound metabolic process
Regulation of macromolecule metabolic process
Regulation of primary metabolic process
Regulation of RNA metabolic process
0
1
2
3
Gene Coverage Percentage (%)
p-value
7.5e-13
5.0e-13
2.5e-13

D

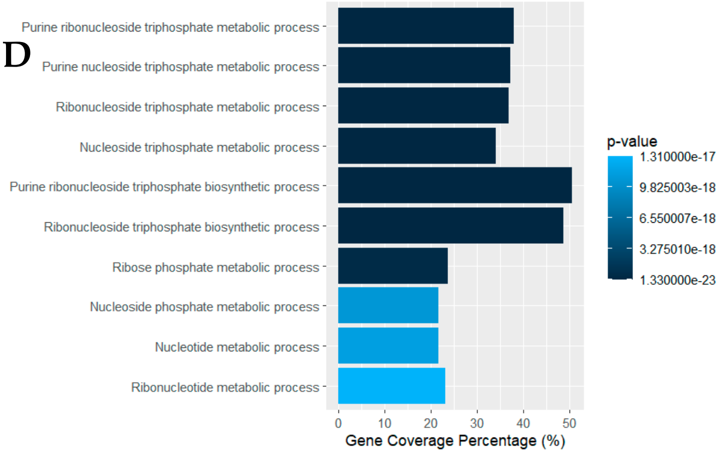
Purine ribonucleoside triphosphate metabolic process
Purine nucleoside triphosphate metabolic process
Ribonucleoside triphosphate metabolic process
Nucleoside triphosphate metabolic process
Purine ribonucleoside triphosphate biosynthetic process
Ribonucleoside triphosphate biosynthetic process
Ribose phosphate metabolic process
Nucleoside phosphate metabolic process
Nucleotide metabolic process
Ribonucleotide metabolic process
0 10 20 30 40 50
Gene Coverage Percentage (%)
p-value
1.310000e-17
9.825003e-18
6.550007e-18
3.275010e-18
1.330000e-23

E

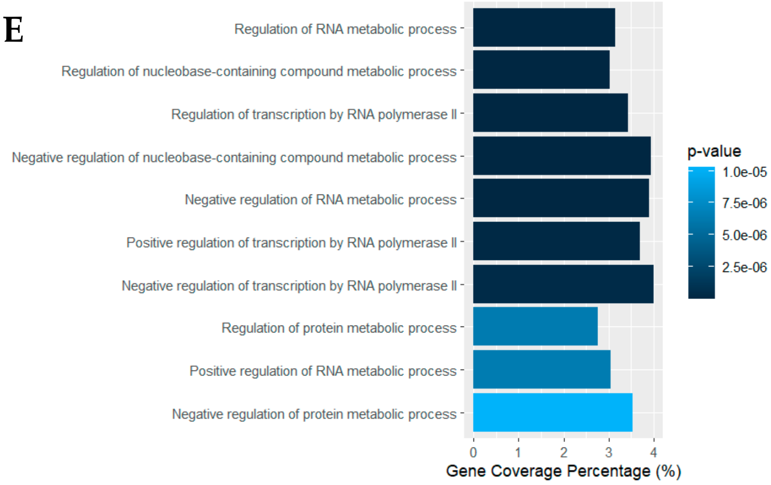
Regulation of RNA metabolic process
Regulation of nucleobase-containing compound metabolic process
Regulation of transcription by RNA polymerase II
Negative regulation of nucleobase-containing compound metabolic process
Negative regulation of RNA metabolic process
Positive regulation of transcription by RNA polymerase II
Negative regulation of transcription by RNA polymerase II
Regulation of protein metabolic process
Positive regulation of RNA metabolic process
Negative regulation of protein metabolic process
0 1 2 3 4
Gene Coverage Percentage (%)
p-value
1.0e-05
7.5e-06
5.0e-06
2.5e-06

F

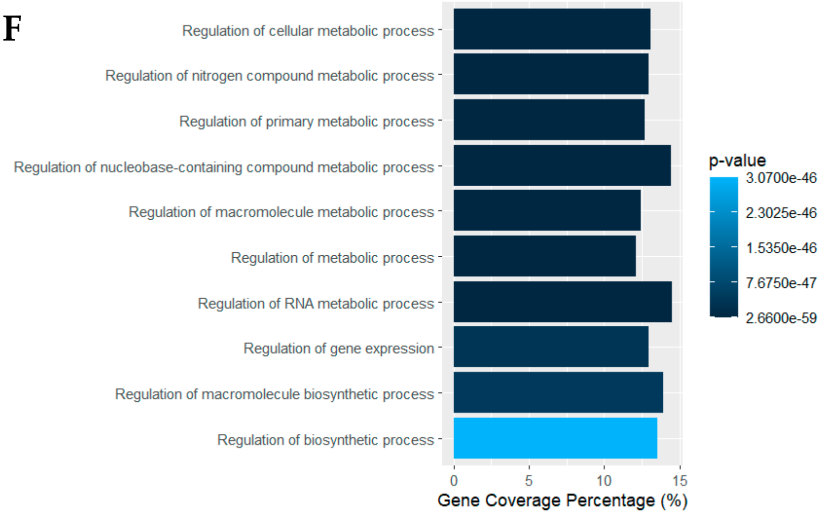
Regulation of cellular metabolic process
Regulation of nitrogen compound metabolic process
Regulation of primary metabolic process
Regulation of nucleobase-containing compound metabolic process
Regulation of macromolecule metabolic process
Regulation of metabolic process
Regulation of RNA metabolic process
Regulation of gene expression
Regulation of macromolecule biosynthetic process
Regulation of biosynthetic process
0 5 10 15
Gene Coverage Percentage (%)
p-value
3.0700e-46
2.3025e-46
1.5350e-46
7.6750e-47
2.6600e-59

G

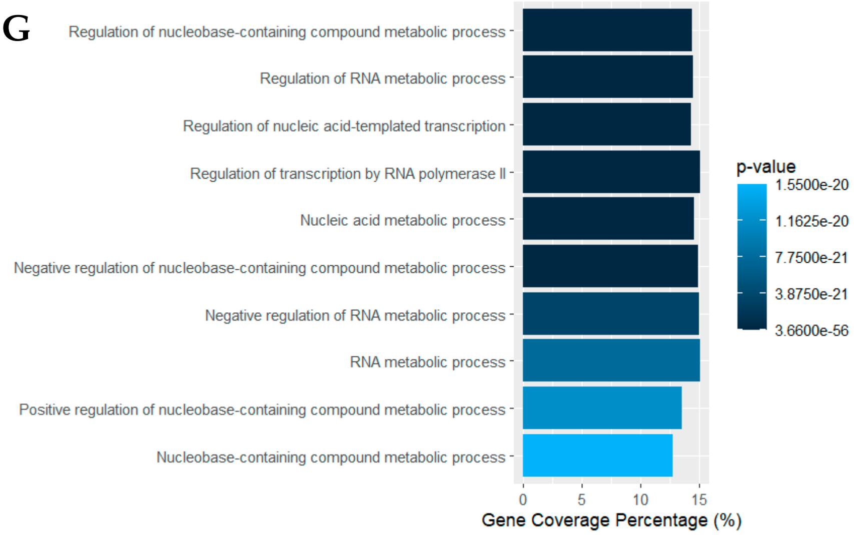

Supplementary Figure S4. Bulk RNA sequencing data processed via pathway analysis.

(**A**) Venn diagram showing numbers of differentially expressed genes unique to either Combi versus Inf, Combi versus Uninf, or similar in both comparisons. ↑, upregulation; ↓, downregulation. The letters **B** to **G** correlate with the following figures to demarcate the set of genes employed for pathway analysis. (**B, C, F**) Untargeted pathway analysis. (**D, E, G**) Targeted analysis of pathways selected by screening for regular expressions containing these terms: “RNA”, “ribo”, “nucle”, “protein”. (**B, D**) Pathway analysis results of uniquely upregulated genes in the Combi versus Inf comparison when contrasted against the Combi versus Uninf comparison. (**C, E**) Pathway analysis results of uniquely upregulated genes in the Combi versus Uninf comparison when contrasted against the Combi versus Inf comparison. (**F, G**) Pathway analysis results of uniquely downregulated genes in the Combi versus Inf comparison when contrasted against the Combi versus Uninf comparison.

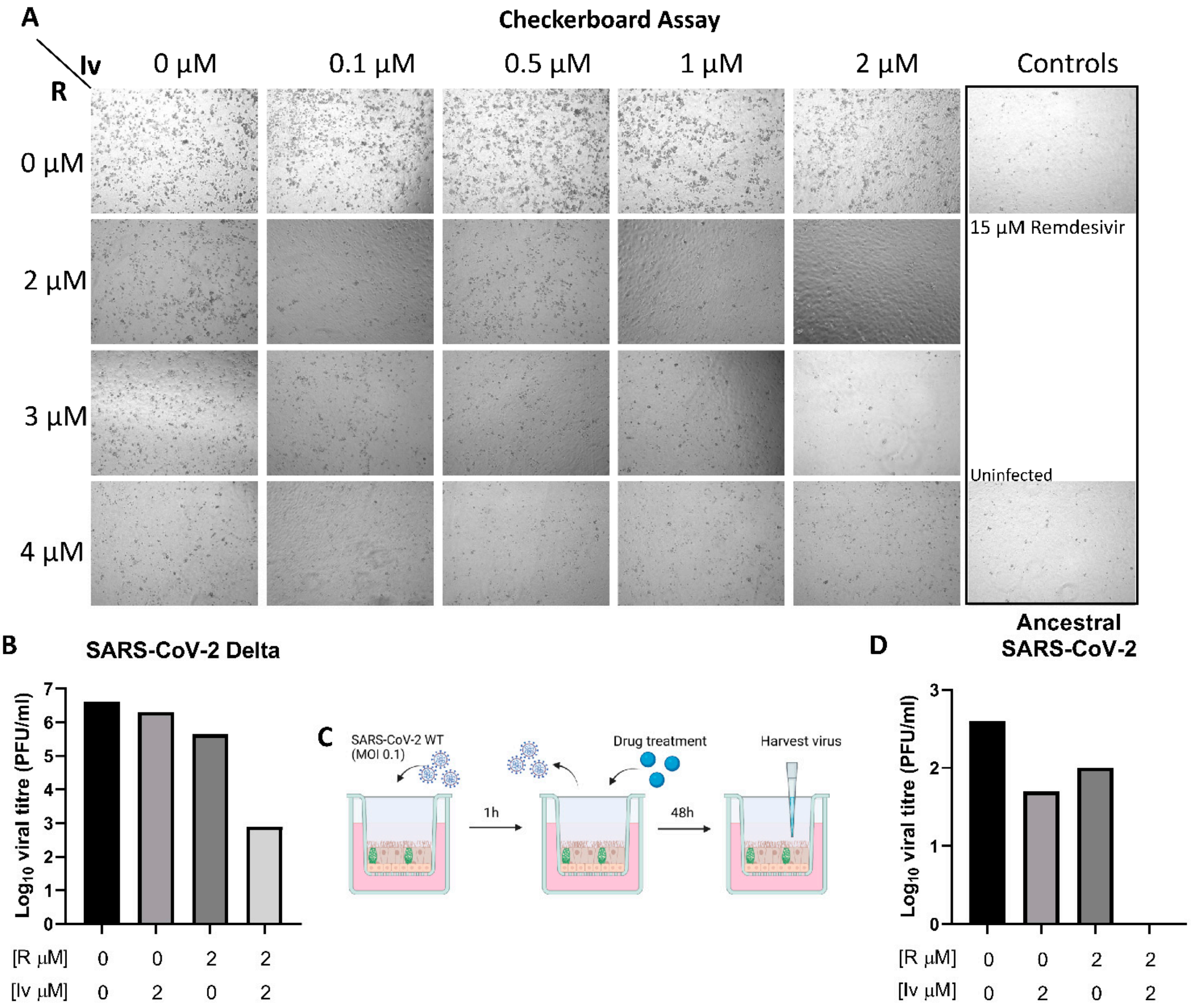

Supplementary Figure S5. Effects of Remdesivir and/or Ivermectin treatments on SARS-CoV-2 infection of VeroE6 cells and human nasal epithelial cell cultures.

(**A**) Representative images of cytopathic effect (CPE) of SARS-CoV-2-infected VeroE6 cells subjected to monotreatments and different combinations of Remdesivir (R) and Ivermectin (Iv) in the checkerboard assay as described in Figure 6B. (**B**) Virus plaque assay results of treatment with Remdesivir and/or Ivermectin in combination or as monotreatment on VeroE6 cells infected at MOI of 0.04 with SARS-CoV-2 Delta variant for 48 h ($n = 1$). (**C**) Procedure for infecting differentiated human nasal epithelial cells in air-liquid interface culture with wild-type (WT) or ancestral SARS-CoV-2. (**D**) Virus plaque assay results of treatment with Remdesivir and/or Ivermectin in combination or as monotreatment on human nasal epithelial cells infected at MOI of 0.1 with ancestral SARS-CoV-2 for 48 h ($n = 1$).

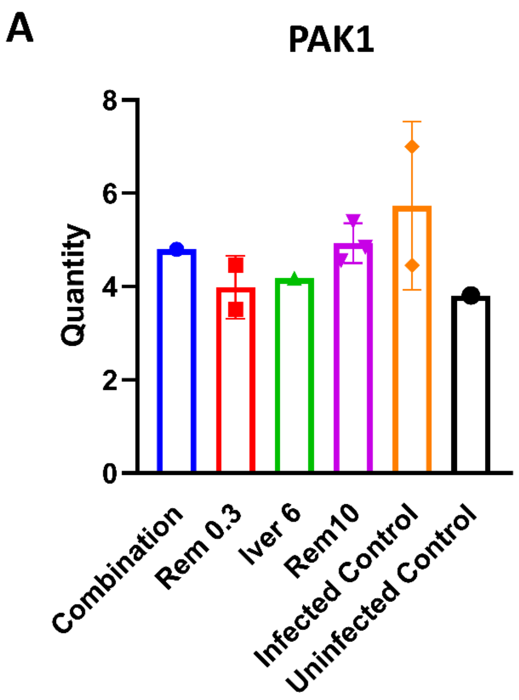

Supplementary Figure S6. Relative quantity of the p21-activated kinase 1 (PAK1) protein in MHV-infected H2.35 cells subjected to different drug treatments as detected by proteomics analysis.

(**A**) The bars indicate the relative quantity of PAK1 protein in the following treatment and control groups: combination treatment with 0.3 µM Remdesivir and 6 µM Ivermectin; 0.3 µM Remdesivir; 6 µM Ivermectin; 10 µM Remdesivir; infected control, and uninfected control (*n* = 2).
